# Abscess of urachal remnants presenting with acute abdomen: a case series

**DOI:** 10.1186/1752-1947-6-226

**Published:** 2012-07-30

**Authors:** Fadl Tazi, Mustapha Ahsaini, Abdelhak Khalouk, Soufiane Mellas, Roos E Stuurman-Wieringa, Mohammed Jamal Elfassi, My Hassan Farih

**Affiliations:** 1 Department of Urology, Hospital University Center Hassan II, Fez, 30000 Morocco; 2 Department of Urology, Reinier de Graaf Gasthuis, P.O. box 5011, Delft, GA, 2600, the Netherlands; 3 Anatomy Laboratory, Faculty of Medicine and Pharmacy of Fez, Fez, 30000 Morocco

**Keywords:** Urachus, Bladder, Neoplasms, Urachal cyst, Urachal remnant, Urachal sinus, Abcess

## Abstract

**Introduction:**

Urachal diseases are rare and may develop from a congenital anomaly in which a persistent or partial reopening of the fetal communication between the bladder and the umbilicus persists. The most frequently reported urachal anomalies in adults are infected urachal cyst and urachal carcinoma. The diagnosis of this entity is not always easy because of the rarity of these diseases and the atypical symptoms at presentation. Imaging techniques, such as ultrasonography and computed tomography have a significant role in recognizing the presence of urachus-derived lesions.

**Cases presentations:**

**Case presentation 1:** A 25-year-old Arab-Berber man presented with a 10-day history of progressive lower abdominal pain accompanied by fever, vomiting, and low urinary tract symptoms to our emergency department. Laboratory data revealed leucocytosis. The diagnosis of an acute peritonitis was made initially. Abdominal ultrasonography revealed a hypoechoic tract from the umbilicus to the abdominal wall, and the diagnosis was rectified (infected urachal remnants). The patient was initially treated with intravenous antibiotics in combination with a percutaneous drainage. Afterwards an extraperitoneal excision of the urachal remnant including a cuff of bladder was performed. The histological analysis did not reveal a tumor of the urachal remnant. Follow-up examinations a few months later showed no abnormality.

**Case presentation 2:** A 35-year-old Arab-Berber man, without prior medical history with one week of abdominal pain, nausea and vomiting, associated with fever but without lower urinary tract symptoms visited our emergency department. Laboratory data revealed leucocytosis. Abdominal ultrasonography was not conclusive. Computed tomography of the abdomen was the key to the investigation and the diagnosis of an abscess of urachal remnants was made. The patient underwent the same choice of medical-surgical treatment as previously described for case one, with a good follow-up result.

**Case presentation 3:** A 22-year-old Arab-Berber man, with no relevant past medical history, presented to our emergency department because of suspected acute surgical abdomen. Physical examination revealed umbilical discharge with erythema and a tender umbilical mass. Abdominal ultrasonography and computed tomography scan confirmed the diagnosis of infected urachal sinus. Initial management was intravenous antibiotics associated with a percutaneous drainage with a good post-operative result, but a few days later, he was readmitted with the same complaint and the decision was made for surgical treatment consisting of excision of the infected urachal sinus. The clinical course was uneventful. Histological examination did not reveal any signs of malignancy.

**Conclusions:**

We describe our clinical observations and an analysis of the existing literature to present the various clinical, radiological, pathological and therapeutic aspects of an abscess of urachal remnants. To the best of our knowledge, this manuscript is an original case report because this atypical presentation is rarely reported in the literature and only a few cases have been described.

## Introduction

The urachus or median umbilical ligament is a fibrous cord that originates from the involution of the allantoic canal. It extends from the bladder dome to the posterior umbilicus. A partial or total defect of obliteration of the urachus channel after the fifth month of gestation can be the origin of urachal abnormalities. The first description was in 1550 by Cabriolus [1] and later a few cases of infected urachal remnants in adulthood were reported in the literature [2-5]. This entity is usually discovered in childhood, but a late onset in adulthood is always possible. In these cases the clinical presentation is highly variable, and makes diagnosis difficult. Therefore, this article describes this rare disease and its possible presentation. It is important to remember the possibility of infected urachal remanants in a patient presenting with an acute surgical abdomen in the emergency department.

## Case presentation

### Case report 1

**Figure 1:**
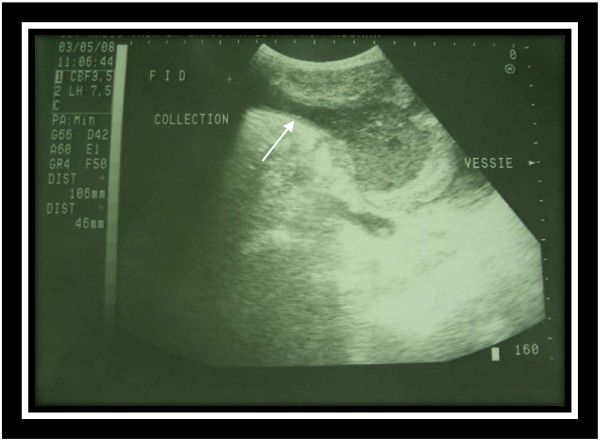
Longitudinal ultrasonography of the lower abdomen shows a heterogeneous urachal collection, extending from the periumbilical region to the dome of the bladder.

A 25-year-old Arab-Berber man, without prior relevant medical history, with lower abdominal pain which had persisted for 10 days presented to our emergency department. The character of the pain was intense and persistent, accompanied with fever, vomiting, and low urinary tract symptoms (for example, polakiuria). On physical examination, he looked tired and his body temperature was 39.5C. Abdominal examination revealed a diffuse tender lower abdomen and midline suprapubic mass measuring 5cm in length. The umbilicus looked normal and no peritoneal signs were elicited. A rectal examination revealed no tenderness and no blood. Laboratory data revealed leucocytosis with 82% neutrophil predominance and a white blood cell count of 13,000/L. Blood biochemistry was normal. Urinalysis was normal and urine culture showed no bacterial growth. A standing abdomen radiography was normal. Abdominal ultrasonography (US) revealed a hypoechoic tract from the umbilicus to the abdominal wall (Figure 1) and a hypoechoic mass with heteroechogenic content between the peritoneum, the muscle layer, and the bladder, without fluid in the peritoneal cavity. The suggested diagnosis was infection of urachal remnants. Treatment was initiated with a broad empirical (amoxiclav + gentamicin) antibiotic accompanied by percutaneous drainage. Cystoscopy was performed but showed no evidence of a bladder anomaly. Finally, extraperitoneal excision of the urachal remnant, including a cuff of bladder, was performed. There were no postoperative complications, and anatomophological analysis did not reveal a tumor of the urachal remnant. The pus culture found a *Proteus mirabilis* infection for which he was treated with ciprofloxacin. At 18 months post-operatively, he was asymptomatic and no abnormalities of the abdominal wall were seen.

### Case report 2

**Figure 2:**
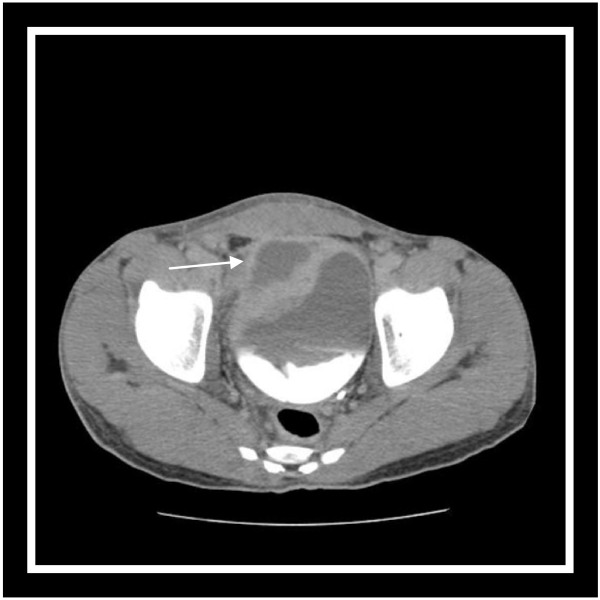
Abdominal computed tomography scan with excretory time shows hypogastric collection above the bladder, with the absence of the communication between the collection and the bladder.

A 35-year-old Arab-Berber man with one week of abdominal pain was admitted to the emergency department. Before coming to our department, the character of his pain was very severe cramping with nausea and vomiting, associated with fever but without lower urinary tract symptoms. He never experienced spontaneous extrusion of pus from his umbilicus. On physical examination, he looked well and his body temperature was 38.5C. The abdomen was soft and tenderness and focal rigidity were found over the umbilical area. No peritoneal signs were elicited and his rectal examination was normal. Laboratory data revealed leucocytosis with 78% neutrophil predominance (white blood cell count of 11,000/L). Blood biochemistry was normal. Urinalysis was normal and non bacteriuric. His standing abdomen x-ray film was normal. Abdominal US revealed a small quantity of fluid in the peritoneal cavity. A computed tomography (CT) of the abdomen was the key to the investigation and showed inflammation with localized abcess formation (65 × 36 × 42mm) from the umbilicus to the dome of bladder, and the surrounding fatty tissue was infiltrated. There was no free intraperitoneal fluid or lymphadenopathy (Figure 2). The suggested diagnosis was abscess of urachal remnants. The choice of medical-surgical treatment was identical to the previously described case.

There were no postoperative complications. On pathological examination, the surgical tissue was mainly composed of fatty and dense fibrous tissues without any cell tumor of the bladder wall. The result of a pus culture showed an *Escherichia coli* infection which was treated with the antibiotic ciprofloxacin. At his follow-up examination two years later he was asymptomatic and had no abnormalities of the abdominal wall.

### Case report 3

**Figure 3:**
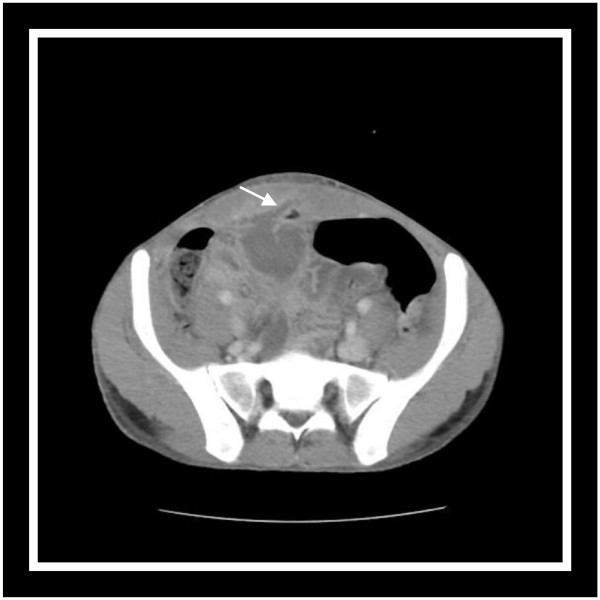
Abdominal computed tomography scan reveals heterogeneous urachal collection communicating with the abdominal wall.

A 22-year-old Arab-Berber man, with no relevant past medical history, presented to our emergency department with a suspected acute surgical abdomen. For some days prior to admission, he had been feeling ill, with a history of fever and abdominal pain without digestive or urinary symptoms. Physical examination revealed an initial temperature of 38.9C, his abdomen was soft and there was umbilical discharge with erythema and a tender umbilical mass. Laboratory tests revealed marked leucocytosis of 24,000/mm3. The urinalysis and renal function were within normal values. Culture of the umbilical discharge revealed a *Klebsiella* pneumonia but a blood culture was negative. His standing abdominal radiography was normal. Abdominal US revealed echoic collection in a midline cavity from the umbilicus to the abdominal wall. CT scan confirmed the diagnosis of infected urachal sinus showing a heterogeneous collection communicating with the umbilicus [Figure 3]. Treatment consisted of intravenous antibiotics (ceftriaxon and gentamicin) associated with a percutaneous drainage.

An emergency cystoscopy was performed and confirmed no evidence of a bladder anomaly. The clinical course was uneventful and he left the hospital. After a few days, he was readmitted with the same complaint, but this time the pain was concentrated around the umbilicus. After a brief evaluation he was treated surgically and the infected urachal sinus was excised using an infra-umbilical midline incision. A follow-up examination at the end of 18 months showed no abnormality. Histological examination did not reveal any signs of malignancy.

## Discussion

**Figure 4:**
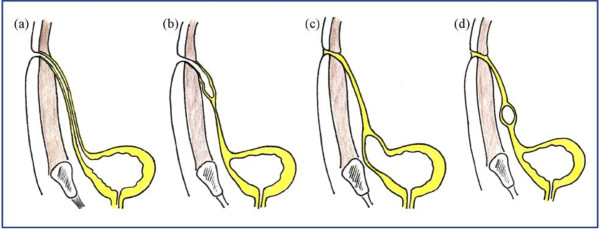
Types of urachal anomalies: (a) patent urachus, (b) urachal sinus, (c) urachal diverticulum, (d) urachal cyst.

The urachus is a normal embryonic remnant of the primitive bladder dome. It generally exists as a fibrous cord extending from the dome of the bladder to the umbilicus. It also occupies the potential midline space between the peritoneum and the transversalis fascia. Urachal diseases can be congenital or acquired, and we support the suggestion that a complete evaluation of the genitourinary tract is warranted in childhood [6,7]. Congenital anomalies occur when the urachus fails to obliterate. The pathology associated with congenital disorder urachus is generally divided into four categories [Figure 4. The first (a) is a patent urachus in which a communication between the bladder and the umbilicus exists. The next category (b) pertains to the umbilical sinus, in which the urachus opens into the umbilicus. Here, drainage from the umbilicus will often be present. The third category (c) is the vesico-urachal diverticulum, in which the urachus has a wide patent opening into the bladder. Urinary complaints are often cited with this type. The last category (d) is the urachal cyst, in which the urachus encompasses a cystlike structure within its length; this last disease state, the urachal cyst, becomes prominent when infection occurs or ruptures of the cyst [8,9]. A recent report showed that children are more likely to have an infected urachal cyst, while adults are more likely to have an infected urachal sinus [7].

Acquired diseases include inflammation and neoplasm. Inflammation occurs more frequently in children and young adults: infected urachal cysts with an onset beyond the fifth decade are quite rare [10,11], although isolated cases of urachal carcinoma in adolescence were reported at the 2007 American Academy of Pediatrics meeting. To the best of our knowledge there has been no association established between urachal remnants in childhood and urachal carcinoma later in life [12].

Malignant degeneration of urachal remnants occurs more frequently in middle-aged and older people [6], and has a clinical course that can be considerably worse than that of primary bladder adenocarcinoma [13].

Firstly, urachal anomalies are rarely observed clinically, with only 8/40,000 admissions to a surgical department, according to Blichert-Toft and Neilson [10]. Secondly, the urachus is located in a clinically silent area, extraperitoneally in the space of Retzius. As a consequence, possible symptoms and clinical signs of inflammation as well as of tumors are in most cases non-specific or delayed, or even absent.

Inflammation as well as the development of an abscess can remain clinically unrecognized for a long time or it can be considered as acute surgical abdomen. Our patients were evaluated by our visceral surgical team at first presentation, therefore, we would not deny this entity if we had a suspected history and detailed exam. Patients may complain of urinary symptoms such as suprapubic pain, dysuria, and/or intermittent episodes of urinary retention [14,15]. General signs of inflammation (such as elevated sedimentation rate, leucocytosis, and fever) may be absent. On urinalysis, bacteriuria and pyuria are absent in more than 80% of cases and urine culture is negative in most cases [14,15]. As a consequence, unless drainage into the umbilicus or bladder occurs, quite often other diseases are suspected, such as Meckel’s diverticulum, acute prostatitis, acute appendicitis, recurrent urinary infections, or abdominal colicky pain of unknown origin [14,15]. Although infection of the cord stump is rare, its potential sequelae such as cellulitis, necrotizing fasciitis, peritonitis, multiple hepatic abscess, septicemia, and possible retroperitoneal abscess may be fatal [16,17].

Urachal lesions are better imaged by US and CT than by any other image modalities. Demonstration of an abscess within the extraperitoneal fat space of the abdominal wall and extension to the umbilicus with or without umbilical discharge is a clue to the diagnosis of urachal abscess [18]. Both US and CT can be used to direct a fine-needle aspiration biopsy [19].

US is usually sufficient to diagnose an infected urachal remnant. Cacciarelli *et al*. described an elliptical, hypoechoic structure in the middle of the anterosuperior surface of the urinary bladder [20]. US showed higher diagnostic accuracy than CT, and the latter has the additional disadvantage of exposure to radiation. Therefore, we recommend US as the first diagnostic tool in the pediatric age group. If this investigation is inconclusive, a CT scan may be used as the second modality, or when malignancy is suspected. Even sophisticated techniques, such as US and CT, can fail to differentiate between inflammatory and neoplastic lesions. Abscesses may have a solid appearance on US [21-23] as well as attenuation values higher than water on CT, thus mimicking a neoplasm [21-23]. On the other hand, tumors may show on CT hypodense central areas due to necrosis, hemorrhage, or mucoid content such that the resulting features may simulate an inflammatory mass [24]. Concerning inflammatory diseases of the urachus, the most common infecting pathogens are *E. coli* and *Proteus*, but a variety of other pathogens can also be found, including *Staphylococcus aureusBacteroidesFusobacterium*, and *Streptococcus iridans*[11,14,15]. Rarely it is secondary to *Actinomycosis*[23], *Aspergillus* or *Tinea corporis*. Occasionally, a chronic inflammatory process can result in the unusual form of a xanthogranulomatous urachitis [22].

The differential diagnosis of urachal abscess should include cellulitis, necrotizing fasciitis, peritonitis, acute appendicitis, hematoma, ventral or umbilical hernia, and tumor lesions especially when it develops into the abdominal wall [25].

For the cases described, the first diagnosis on admission was peritonitis. However, it is important to think of the entity urachal remnants when no other origin is found, especially in young patients.

The management of the symptomatic urachal remnants depends on the age of the patient when the disease is first discovered. Generally, in childhood surgical intervention should be avoided for patients younger than one year because the remnant might spontaneously disappear. Surgical resection should be restricted to patients with multiple symptomatic episodes who are older than one year. Therefore, only a few cases should require surgical resection. In addition, most of the patients with asymptomatic urachal remnants do not require regular follow-up. Continuous observation with periodic ultrasound examinations is not necessary for asymptomatic cases [26].

However, treatment of infectious urachal remnants in adulthood should include antibiotic therapy for the acute infection followed by primary or secondary excision after draining the abscess cavity. Blichert-Toft and Nielson [10,27-29] reported that up to 31% of infected cysts recurred when not excised in the absence of infection; urachal cyst excision provides the most benign postoperative course. It is generally recommended that all urachal remnants should be excised to avoid recurrent disease as happened in our third patient presentation and because of possible malignant transformation later in life [30]. However, when infection is present, management by preoperative percutaneous drainage and subsequent elective excision may represent the most effective surgical option. Although the condition is not well defined, the possibility of adenocarcinoma in an incompletely resected specimen led to the practice of radical excision of the urachal remnant. Radical excision requires removing all the structures within the umbilicovesical fascia [10,27,28]; including the urachus and each medial umbilical ligament, as well as the associated peritoneum from the umbilicus to the bladder dome. For benign lesions that do not communicate with the umbilicus or bladder there is no consensus on whether the umbilicus and a bladder cuff should be resected routinely [27-29,31]. Most reports of urachal cyst excision [27,28,32] do not mention umbilical resection [28,32,33].

Traditional surgical excision of an urachal remnant involves a transverse or midline infra-umbilical incision. To minimize the morbidity of surgery (for example, postoperative pain and prolonged convalescence), the laparoscopic approach for resection of the urachus was first introduced by Trondsen *et al*. [30] Since then some other teams have published their series of a laparoscopic approach [27,28,31,34-36].

Each case was technically feasible with acceptable operative time but no quantitative assessment of outcome and morbidity was provided [33,37]. Using three or four trocars 12mm or less we adhered to the basic surgical principles of urachal surgery in each case [33], such as medial umbilical ligaments with or without a bladder cuff. This technique offers many advantages, such as short duration of hospital stay (2.75 days) and brief recovery with a mean return to normal activity in 11 days [33,37]. In addition, the laparoscopic approach provides an improved cosmetic result. Recently, Maciej Patrzyk *et al*. [38] have suggested and described a minimally invasive technique: single incision laparoscopic surgery (SILS), with the advantage of a better cosmetic result that can be adopted as an optional laparoscopic approach in specialized centers.

## Conclusions

In summary, urachal abscess may only present with abdominal pain without obvious erythematous periumbilical tissue or exudates in adults. Despite the low incidence in adult patients, it should not be ignored in the differential diagnosis of abdominal pain. History taking and detailed physical examination may help in early diagnosis. US and CT scan are the gold standard diagnostic tools for suspected cases of urachal lesions.

Treatment of the infected urachal remnants in adulthood should consist of antibiotic treatment combined with adequate drainage and later followed by total excision of the remnant including resection of the bladder cuff. This strategy seems to be the most effective treatment option.

The laparoscopic approach appears to be a safe and effective alternative to open surgery in the management of urachal remnants with recurrent infections in both infants and adults. It provides easier access to the bladder dome in contrast to classical open surgical incisions. In addition it permits an acceptable excision of the lesion without producing marked scarring. It yields good long-term cosmetic results.

### Consent

Written informed consent was obtained from the patients for publication of this manuscript and any accompanying images. A copy of the written consent is available for review by the Editor-in-Chief of this journal.

## Competing interests

The authors declare that they have no competing interests.

## Authors’ contributions

FT and MA are the principal authors and major contributors in writing the manuscript. AK and SM analyzed and interpreted the patient data and reviews of the literature. MJE and MHF read and corrected the manuscript. RSW contributed to the writing and correction of this paper. All authors read and approved the final manuscript.
